# An Amorphous Native Oxide Shell for High Bias‐Stress Stability Nanowire Synaptic Transistor

**DOI:** 10.1002/advs.202302516

**Published:** 2023-09-28

**Authors:** Xinming Zhuang, Zixu Sa, Jie Zhang, Mingxu Wang, Mingsheng Xu, Fengjing Liu, Kepeng Song, Tao He, Feng Chen, Zai‐xing Yang

**Affiliations:** ^1^ School of Physics State Key Laboratory of Crystal Materials Shandong University Jinan 250100 P. R. China; ^2^ School of Microelectronics Shandong University Jinan 250100 P. R. China; ^3^ School of Chemistry and Chemical Engineering Shandong University Jinan 250100 P. R. China

**Keywords:** bias‐stress stability, field‐effect‐transistors, GaSb nanowires, native oxide shells, synaptic transistors

## Abstract

The inhomogeneous native oxide shells on the surfaces of III–V group semiconductors typically yield inferior and unstable electrical properties metrics, challenging the development of next‐generation integrated circuits. Herein, the native GaO*_x_* shells are profitably utilized by a simple in‐situ thermal annealing process to achieve high‐performance GaSb nanowires (NWs) field‐effect‐transistors (FETs) with excellent bias‐stress stability and synaptic behaviors. By an optimal annealing time of 5 min, the as‐constructed GaSb NW FET demonstrates excellent stability with a minimal shift of transfer curve (Δ*V*_th_ ≈ 0.54 V) under a 60 min gate bias, which is far more stable than that of pristine GaSb NW FET (Δ*V*_th_ ≈ 8.2 V). When the high bias‐stress stability NW FET is used as the chargeable‐dielectric free synaptic transistor, the typical synaptic behaviors, such as short‐term plasticity, long‐term plasticity, spike‐time‐dependent plasticity, and reliable learning stability are demonstrated successfully through the voltage tests. The mobile oxygen ion in the native GaO*_x_* shell strongly offsets the trapping states and leads to enhanced bias‐stress stability and charge retention capability for synaptic behaviors. This work provides a new way of utilizing the native oxide shell to realize stable FET for chargeable‐dielectric free neuromorphic computing systems.

## Graphical abstract

A tunable approach is reported first to manipulate the amorphous native GaO*_x_* shells of GaSb NWs via a simple in situ annealing process. Furthermore, these native GaO*_x_* shells are studied systematically for improving the bias‐stress stability of the GaSb NWs FETs and broadening the application in chargeable‐dielectric free synaptic transistors with typical synaptic behaviors and reliable learning stability.

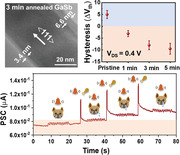

## 1 Introduction

III–V group semiconductors, such as GaSb, GaAs, InSb, InAs, InP, and InGaAs, possess the advantages of superior carrier mobility and tunable bandgap, composition, structural configuration, which have been considered promising candidates for next‐generation electronics and optoelectronics.^[^ ^1^, ^2^, ^3^, ^4^, ^5^, ^6^ ^]^ However, when these pristine semiconductors are exposed to the atmosphere, the highly reactive dangling bonds will promote the formation of native surface oxides, resulting in unwanted interfacial defects and surface states.^[^ ^7^, ^8^ ^]^ The unwanted interfacial defects usually act as charge trapping centers, resulting in capacitance‐frequency dispersion, *I*–*V* hysteresis, and bias‐stress instability when III–V group semiconductors are constructed into electronic devices.^[^ ^9^, ^10^, ^11^, ^12^ ^]^ As pointed out by Lan et al., the native oxide shells on the surfaces of InAs nanowires (NWs) acted as surface charge trappings, contributing to the gate bias‐stress instability and hysteresis in the as‐constructed field‐effect‐transistors (FETs).^[^ ^13^, ^14^ ^]^ Furthermore, the unwanted surface states, originating from the native oxide shells, will introduce the interface‐trap states, which will cause a relatively strong Fermi‐level pinning effect.^[^ ^15^ ^]^ Take GaAs as an example, the high density of surface states pin the surface Fermi level within the band gap, which gives rise to the metal‐independent variation of *I*–*V* characteristics and inferior operation stability of electronic devices.^[^ ^16^ ^]^

It's worth noting that, enlightened by the oxidation of SiO_2_ on the surface of Si, several reports have recently tried to utilize the native oxide shells in an opposite profitable way.^[^ ^17^, ^18^ ^]^ This is because the oxidized native shell can serve as a protective layer against contamination and also as a natural gate dielectric in FETs, owing to its insulating property and excellent interface quality. For example, owing to the high dielectric constant, the atomically sharp and chemically clean semiconductor‐oxide interface of native oxide dielectric shell, Peng et al. have successfully realized high‐performance Bi_2_O_2_Se FETs, FinFETs, as well as inverter circuits, recently.^[^ ^19^, ^20^ ^]^ Besides, more typical devices, such as flexible FETs, resistive switching random access memories, vertical light emitters, and photodetectors have also been obtained by utilizing the native oxide shells.^[^ ^21^ ^]^ It becomes possible to manipulate the native oxide shell of III–V group semiconductors. Fortunately, as revealed in the amorphous GaO*_x_* thin film, an applied gate voltage can cause the appreciable polarization of oxygen ions, contributing to the suppression of trapping density and nonvolatile memristive behavior.^[^ ^22^, ^23^, ^24^ ^]^ This inspirits the implementation of Ga‐based III–V semiconductor devices for stable electronics, optoelectronics, and neuromorphic electronics by manipulating the native oxide shells. It is worth pointing out that these native GaO*_x_* shell‐based synaptic FETs can effectively avoid the use of a chargeable dielectric (including ferroelectrics,^[^ ^25^, ^26^ ^]^ electrolytes,^[^ ^27^ ^]^ ionic gels,^[^ ^28^ ^]^ and floating gate^[^ ^29^ ^]^), thus not only cutting down the manufacturing costs, but also providing an atomically sharp and chemically clean interface for reducing challenges of additional dielectric affecting the quality of the top channel.^[^ ^30^ ^]^

As a typical Ga‐based III–V semiconductor, GaSb NW has attracted considerable interest in versatile electronics and optoelectronics due to the narrow direct bandgap and high hole mobility.^[^ ^31^, ^32^ ^]^ It is noteworthy that the rich dangling bonds on the surface of GaSb NW inevitably contribute to the forming of native oxide shells of GaO*_x_*.^[^ ^33^ ^]^ In this case, it is ingenious and strongly desired to explore reliable GaSb NW FETs and synaptic transistors by purely manipulating the native oxide shell of GaO*_x_*. In this contribution, we demonstrate a facile approach to manipulate the amorphous native oxide shell on the surface of GaSb NW by using an in‐situ annealing process. By carefully manipulating the thickness of the native GaO*_x_* shell, the as‐constructed GaSb NW FET demonstrates excellent bias‐stress stability with a minimal *V*_th_ shift of ≈0.54 V under 60 min voltage bias (*V*_GS_ = −10 V and *V*_DS_ = 0.4 V). Furthermore, the typical synaptic behaviors and Pavlovian conditioning are demonstrated successfully by adopting the bias‐stress stability NW FET. Our results show that the enhanced bias‐stress stability and synaptic properties are attributed to the migration of oxygen ions in the amorphous GaO*_x_* shell, which suppresses the hole‐trapping states and leads to the chargeable capability for synaptic behaviors. Utilizing the ionic migration characteristic in the native oxide shell of III–V group semiconductors paves a new way for bias‐stress stability electronic devices and advanced stable neuromorphic systems.

## 2 Results and Discussion

### 2.1 Native Oxide Shelled GaSb NWs Growth and Characterization

**Figure 1:**
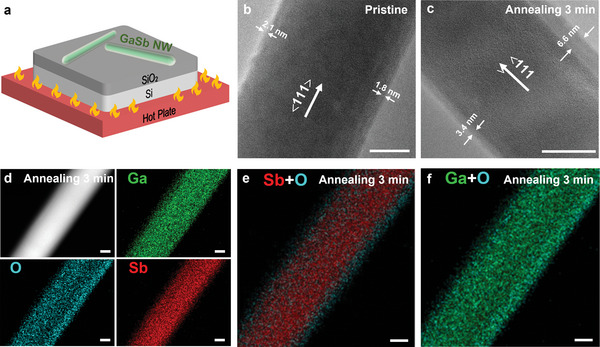
Native oxide shelled GaSb NWs growth and characterization. a) Schematic of thermal annealing oxidation process. HRTEM images of b) pristine, and c) 3 min annealed GaSb NW in this study. EDS elemental mapping images of d) individual elements (Ga, O, and Sb), e) Sb+O elements, and f) Ga+O elements, respectively, of 3 min annealed GaSb NW. All the scale bars are 20 nm.

GaSb NWs are prepared by adopting the surfactant‐assisted solid‐source chemical vapor deposition (CVD) method and the native oxide shelled GaSb NW are prepared by using an in‐situ annealing process on a hot plate at 300 °C for 0, 1, 3, and 5 min (**Figure** **1**a). The thicknesses of native oxide shells are controlled easily by the annealing time. Details regarding the preparation processes can be found in the Experimental Section. Scanning electron microscopy (SEM) is first performed on the as‐prepared NWs to monitor the surface morphology. It is seen that the NWs are dense, long, and thin uniform in diameter along the axial direction and the thermal annealing processes do not obviously change the morphology (Figure S1a–d, Supporting Information). As shown in Figure 1b,c and Figure S1e,f (Supporting Information) of the high‐resolution transmission electron microscopy (HRTEM) images, all GaSb NWs show core‐shell nanostructures, along with clear contrasts between the cores and shells. The thicknesses of native oxide shells around the GaSb cores steadily increase from 1.95 ± 0.21 to 4.25 ± 2.47, 5.00 ± 2.26, and 5.75 ± 1.48 nm, as the thermal annealing time increases from 0 to 1, 3, and 5 min, respectively. The increased thickness error in annealed GaSb NWs may originate from the differences in oxygen concentration and annealing temperature at the upper and bottom sides of GaSb NWs. To further identify the compositions of the core and shell, energy dispersive spectroscopy (EDS) elemental mapping is conducted under scanning TEM mode (Figure 1d–f; Figure S2, Supporting Information). As shown in Figure S2a,b (Supporting Information), there is no clear distinction between Ga, O, and Sb in pristine GaSb NW, indicating that the native oxide shell is pretty thin in terms of resolution. However, upon 3 min annealed GaSb NW, the Sb element is clearly dominated in the core, and the Ga+O elements are distributed in the whole NW, indicating the formation of GaO*_x_* on the surface of GaSb NW (Figure 1d–f). Further increasing the thermal annealing time will controllably broaden the thickness of the GaO*_x_* shell. Meanwhile, X‐ray diffraction (XRD) measurements (Figure S3, Supporting Information) show that only the diffraction peaks of zinc blende GaSb (JCPDS Card No. 07‐0215) are observed,^[^ ^34^ ^]^ which confirms that the GaO*_x_* shell is essentially amorphous. In short, the results show that the simple thermal annealing process could successfully manipulate the native oxide shells (GaO*_x_*) of GaSb NWs with controllable thicknesses.

### 2.2 Response Characteristics of the Indicated Native Oxide Shelled GaSb NWs FETs

**Figure 2:**
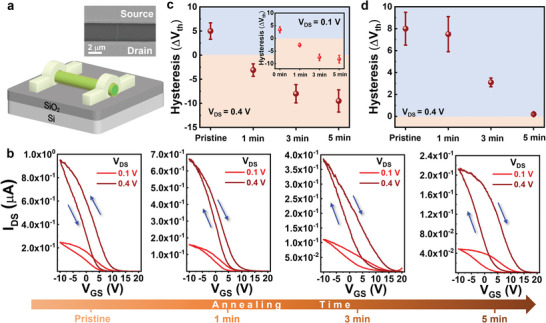
Response characteristics of the indicated native oxide‐shelled GaSb NWs FETs. a) Structure of the native oxide‐shelled GaSb NWs FETs. b) Representative transfer characteristics of the FETs based on GaSb NWs under various annealing times. Hystereses of Δ*V*_th_ statistics of these indicated GaSb NWs FETs under c) the fast and d) slow gate sweeping rate. Statistics from ≥10 devices.

After that, the back‐gated FETs based on the indicated GaSb NWs are fabricated to investigate the effects of native oxide shells on response characteristics. Fabrication details can be found in the Experimental Section and the device structure is shown in **Figure** **2**a. Figure 2b and Figure S4 (Supporting Information) show representative transfer and output characteristics, respectively (voltage sweep rate = 1.1 V⋅s^−1^), inferring the typical ohmic contacts and *p*‐type conductive behaviors even after the thermal annealing oxidation. For the transfer curve, the FET comprised by the pristine GaSb NW exhibits representative anticlockwise hysteresis, which represents the drain current (*I*_DS_) in the back‐sweep curve is always lower than that in a forward‐sweep curve in the case of a continuous *V*_GS_ bias sweep from 20 to −10 V, and then back to 20 V. This is due to more holes trapped at the GaSb/SiO_2_ interface in the back‐sweep curve, indicating the abundance surface traps on the surfaces of NWs.^[^ ^35^ ^]^ Interestingly, upon thermal annealed NW FETs, anticlockwise hysteresis changes to clockwise hysteresis and then steadily increases as the thermal annealing time increases from 1 to 5 min. The hystereses of Δ*V*_th_ (*V*_th_ subtraction in forward‐sweep curve and back‐sweep curve) under *V*_DS_ of 0.1 and 0.4 V are calculated for quantitative analysis in Figure 2c. Positive and negative hystereses mean anticlockwise and clockwise hystereses, respectively. For the pristine GaSb NW FET, the anticlockwise hystereses exhibit the steady Δ*V*_th_ of 3.4 ± 1.2 V (*V*_DS_ = 0.1 V) and 5.0 ± 1.7 V (*V*_DS_ = 0.4 V). The relatively large hystereses errors result from the complex and abundant surface traps on the surfaces of NWs. With the thermal annealing time increasing from 1 to 3 and 5 min, the clockwise hystereses under *V*_DS_ = 0.1 V steadily increase from −2.6 ± 0.7 to −7.5 ± 1.3 and −8.2 ± 1.5 V, respectively. A similar hysteresis trend is observed for a higher *V*_DS_ of 0.4 V. The clockwise hystereses steadily increase from −3.1 ± 1.3 V (annealing 1 min) to −8.0 ± 1.9 V (annealing 3 min) and −9.5 ± 2.3 V (annealing 5 min). The clockwise hysteresis in *p*‐type FET indicates the chargeable effect.^[^ ^30^ ^]^ This chargeable effect is most likely from the native GaO*_x_* shell of GaSb NW.^[^ ^22^ ^]^ It is well‐known that substantial hysteresis is highly dependent on the sweeping rate.^[^ ^14^ ^]^ Thus, we further characterize the hystereses of the present pristine GaSb NW FET with various sweeping rates of 1.1 V⋅s^−1^ (fast), 0.3 V⋅s^−1^ (medium), and 0.07 V⋅s^−1^ (slow) under *V*_DS_ = 0.4 V, as shown in Figure S5a–c (Supporting Information). As a result, the enhanced anticlockwise hystereses of 5.0 ± 1.7, 6.1 ± 1.4, and 8.0 V ± 1.5 V are observed in the transfer curves, which are attributed to the dynamic charge trapping effects caused by the gate bias stress. On the other hand, after the thermal annealing process, the significantly reduced anticlockwise hystereses of 7.5 ± 1.6 V (annealing 1 min), 3.1 ± 0.4 V (annealing 3 min), and ≈0.1 V (annealing 5 min) are obtained under the long time gate bias stress (Figure 2d; Figure S5d–f, Supporting Information), implying the existence of chargeable compensatory effect during the operation process. Performance metrics of the indicated GaSb NW FETs are summarized in **Table** **1**. Due to the amorphous GaO*_x_* shell inhibits the conductive channel at the interface between GaSb NW and the dielectric layer, the mobility, and *I*_DS_ slightly degrades as the thermal annealing time increases. Anyway, the negligible *I*–*V* hysteresis indicates the annealed NW FETs will possess an improved gate bias‐stress stability.

**Table 1:** Performance metrics of the indicated GaSb NW FETs.

Device^a)^	Hysteresis [V]	Mobility [cm^2^/V⋅s]	*V*_th_ [V]	*I*_on_ [µA]	*I*_on_/*I*_off_
Pristine	5.0±1.7	239±38	8±2.2	0.95±0.21	≈10^3^
Annealing 1 min	−3.1±1.3	162±27	5.2±1.8	0.66±0.18	≈10^3^
Annealing 3 min	−8.0±1.9	106±16	5.0±1.5	0.38±0.12	≈10^3^
Annealing 5 min	−9.5±2.3	59±12	3.3±1.1	0.21±0.08	≈10^3^

^a)^ Hysteresis, *V*_th_, mobility, *I*_on_, and *I*_on_/*I*_off_ were extracted from the transfer characteristic curve under *V*_DS_ = 0.4 V. Positive and negative hysteresis means anticlockwise and clockwise hysteresis, respectively. *V*_th_ and mobility were calculated from the forward transfer curve. Statistics from ≥10 devices.

### 2.3 Gate Bias‐Stress Stability of the Indicated Native Oxide Shelled GaSb NWs FETs

**Figure 3:**
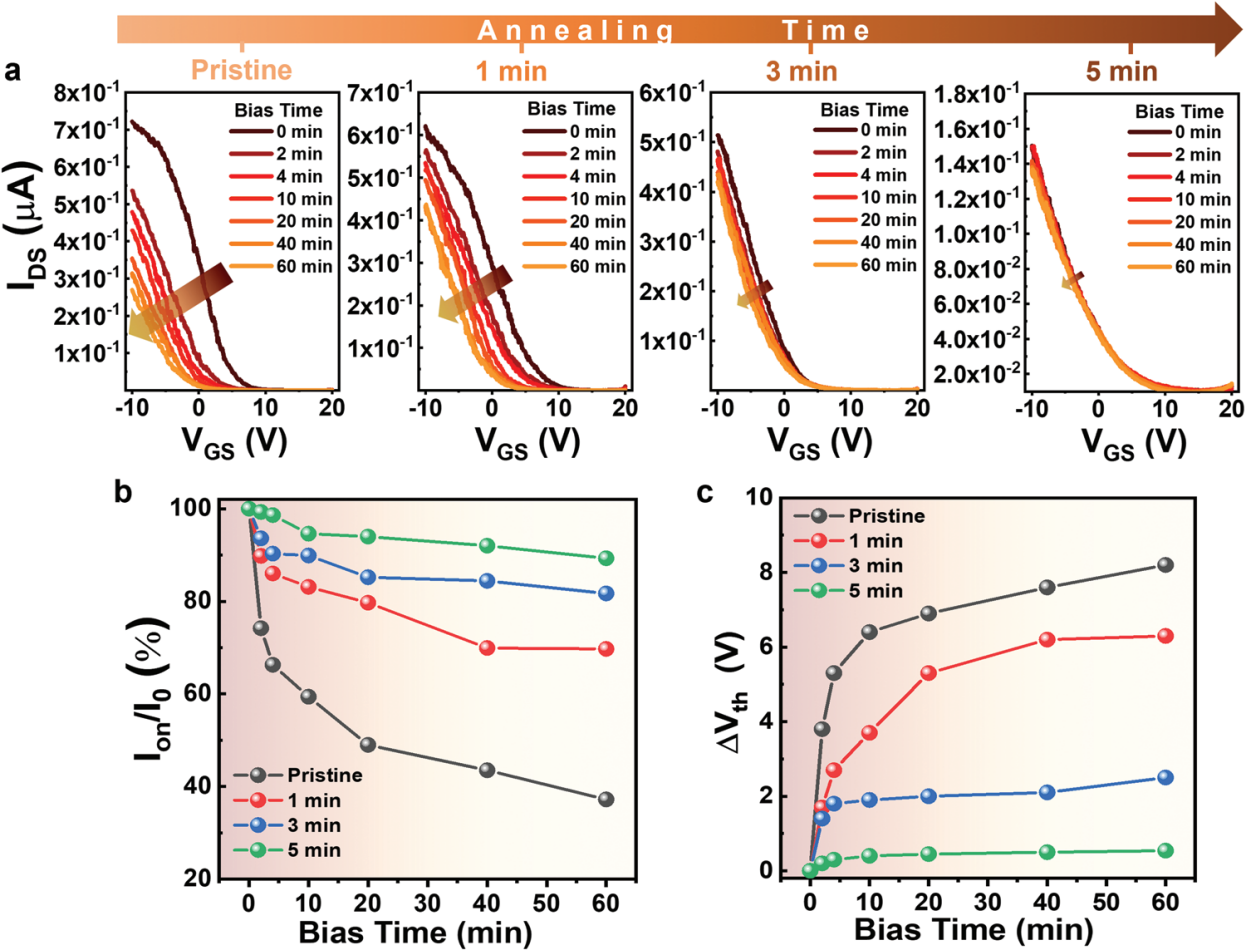
Gate bias‐stress stability of the indicated native oxide‐shelled GaSb NWs FETs. a) Bias stress measurements of representative FETs based on pristine, 1, 3, and 5 min annealed GaSb NWs. Changing ratio of b) *I*_on_ versus initial current *I*_0_ and threshold voltage shifts of c) Δ*V*_th_ as a function of bias time.

In practical applications, such as backplanes for display, integrated circuits, and radio‐frequency identifications, long‐term bias stress is commonly constantly applied to FETs.^[^ ^36^ ^]^ Therefore, bias‐stress stability is an extremely important parameter for identifying the quality of FETs. Based on the above results, gate bias‐stress stability tests with the bias of *V*_GS_ = −10 V and *V*_DS_ = 0.4 V of indicated native oxide shelled GaSb NWs FETs are conducted to assess the amorphous shell of GaO*_x_* behavior and compared with the static *I–V* characteristics shown above. As presented in **Figure** **3**a, all the FETs show a decreasing *I*_on_ and a negative shifting *V*_th_ along with prolonged bias time. Here, the *I*_on_ variation (*I*_on_/*I*_0_) and Δ*V*_th_ are monitored for 60 min to quantitatively describe the stable properties of these native oxide‐shelled GaSb NWs FETs (Figure 3b,c). Thus, the *I*_on_ of the pristine FET decreases from 0.72 µA (0 min) to 0.27 µA (60 min), retaining only 37.2% of the original value during the bias stress test. This corresponds to *V*_th_, shifted from 5.1 V (0 min) to −3.1 V (60 min), yielding a Δ*V*_th_ of 8.2 V. The negative shifting *V*_th_ is ascribable to the considerable trappings accumulation at the interface of GaSb NW and dielectric. These results are consistent with the huge anticlockwise hysteresis characteristics in pristine FET under a long‐time *V*_GS_ sweep rate. However, for 1 and 3 min annealed FETs, *I*_on_ is far more stable, retaining 69.7% and 81.7% of the original value, with Δ*V*_th_ of 6.3 and 2.5 V, respectively. It is witnessed that the 5 min annealed FET shows the smallest *I*_on_ variation (89.3% retaining) and *V*_th_ shift (0.54 V) under the 60 min gate bias stress. The density of trapped charges (*N*_t_) is further estimated based on the formula as follows: *N*_t_ = *C*_ox_⋅Δ*V*_th_/*q*, where *C*_ox_ and *q* denote the capacitance and unit charge, respectively.^[^ ^37^ ^]^ The calculated newly generated trapping density of the 5 min annealed FET is ≈2.4 × 10^11^ cm^−2^, which is over 15× lower than that of pristine FET (3.7 × 10^12^ cm^−2^). This tiny newly generated trapping density is consistent with the negligible *I–V* hysteresis, meaning that trapping states at the interface are effectively eliminated by the native amorphous GaO*_x_* shell.

### 2.4 Synaptic Behaviors Mimicked in the Native Oxide Shelled GaSb NWs FETs

**Figure 4:**
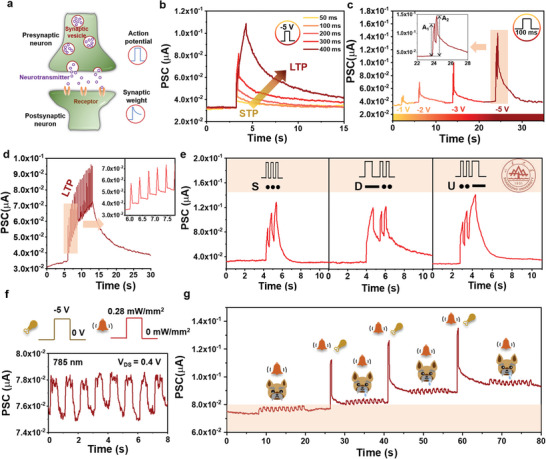
Synaptic behaviors mimicked in the native oxide‐shelled GaSb NWs FETs. a) Schematic illustration of the biological synapse. b) PSC of the synaptic transistor with the 5 min annealed GaSb NWs, demonstrating the typical transformation from STP to LTP. c) PPF behaviors of 5 min annealed GaSb NWs synaptic transistor. d) The ability of LTP under 21 consecutive *V*_GS_ pulses (*V*_GS_ = −5 V, width = 100 ms, interval time = 300 ms). e) PSCs evoked by a series of *V*_GS_ pulses (−5 V) with the International Morse code of “SDU (Shandong University)”. The pulse duration of dot and dash patterns are 300 and 1200 ms, respectively. f) The combination of electrical and infrared pulses and response in the infrared response of synaptic transistors. g) Mimicking Pavlovian conditioning through electrical and infrared pulses.

In a biological neural network, the presynaptic neuron typically receives chemical signals and induces the electric impulse in the postsynaptic neuron through a neurotransmitter (**Figure** **4**a).^[^ ^38^ ^]^ To match the power consumption of human brain synapses (≈fJ per synaptic event), the action potential of a presynaptic neuron is usually on the order of milliseconds.^[^ ^39^ ^]^ In this case, the clockwise hysteresis in a short time sweep curve, seeming chargeable effect, indicates that the annealed FET has the potential to be used as a synaptic transistor. Herein, as a proof of concept, the negative voltage spike (*V*_GS_ pulse) and the resulting enhanced positive drain current are corresponded to the presynaptic input and the postsynaptic current (PSC), respectively. The short‐term plasticity (STP), which is activated by transient stimuli and then decays to the initial value quickly, is first emulated by GaSb NWs FETs.^[^ ^38^, ^40^ ^]^ Notably, the pristine GaSb NW FET shows no obvious decayed characteristics when the presynaptic spike increased from −1 to −5 V, indicating that the pristine device is volatile (Figure S6, Supporting Information). In contrast, 5 min annealed GaSb NW FET exhibits significant nonvolatility when the presynaptic voltages increase from −1 to −5 V, likely promoted by the amorphous GaO*_x_* shell (Figure S7, Supporting Information). In contrast to STP, long‐term plasticity (LTP) plays an important role in memory and learning by altering the synaptic weight more persistently.^[^ ^38^ ^]^ To explore the process of LTP, a series of presynaptic voltages with fixed pulse amplitude (−5 V) and various durations (from 50 to 400 ms) are applied to the 5 min annealed GaSb NW FET. As shown in Figure 4b, the nonvolatility of the 5 min annealed GaSb NW FET increases gradually when the duration increases from 50 to 400 ms, designating the transition from STP to LTP. Furthermore, paired‐pulse facilitation (PPF), which demonstrates that synapses can inherit and adjust the weight based on the time interval between paired spikes, is closely related to the synaptic capability of executing complicated computing.^[^ ^41^ ^]^ As shown in Figure 4c, the PSC triggered by the second *V*_GS_ pulse (Δ*t* = 100 ms) is obviously larger than that induced by the first pulse, which is a characteristic indication of the PPF behavior. In this case, the PSC is triggered by a pair of gate pulses (*V*_GS_ = −5 V for 100 ms, and the pulse interval ranges from 100 to 600 ms). As shown in Figure S8 (Supporting Information), the PPF index decreases as the pulse interval increases with a maximum PPF index value of 121% when Δ*t* is 100 ms. The pulse interval‐dependent decay of the PPF index demonstrates the successful simulation of biological synapses. Apart from the temporally related plasticity discussed above, consecutive multiple (21) *V*_GS_ pulses (*V*_GS_ = −5 V, width = 100 ms, interval time = 300 ms) are successfully demonstrated in the native oxide shelled GaSb NW FET with a result of a permanent change in synaptic connectivity, suggesting a more active role in precisely defining their synaptic weight according to the different spike intensity (Figure 4d).

The simulation of spatiotemporally correlated signal processing is of great significance for a neuromorphic computing system. Herein, the international Morse codes are used as patterned *V*_GS_ pulse signals to further investigate the practical potential of native GaO*_x_*‐shelled GaSb NWs FETs. It is explicit that the synaptic transistor adopted by the native GaO*_x_* shelled GaSb NW FET could accurately demonstrate a reliable PSC response for every letter of S, D, and U (Figure 4e), which indicates the outstanding STP and LTP characteristics and exhibits potential for the radiocommunication application. Furthermore, the classical Pavlovian conditioning, which is known as simple associative learning, is further illustrated for practical utilization of the as‐studied synaptic transistor.^[^ ^42^ ^]^ Here, the food and bell ringing are referred to as unconditional stimulus (US) and conditional stimulus (CS), respectively. With the time‐dependent LTP, the electrical *V*_GS_ pulses can emulate food to activate salivation, defined as the unconditioned response (UR). Meanwhile, owing to the infrared light response of GaSb NW (details response is shown in Figure 4f), the stimulation of 785 nm light pulses can emulate bell to stimulate the conditioned response (CR). During the synergistic photoelectric modulation with detection parameters of responsivity (74.67 A/W) and detectivity (1.87 × 10^10^ Jones), the period of CS is comparable with US to achieve the associative learning process. As depicted in Figure 4g, when the bell ring is imitated by a series of infrared pulses for the first time, the salivation simulated by PSC is not activated (PSC < 80 nA). In contrast, after combining the stimulations of the bell and food, the PSC would increase above the threshold and the dog will salivate after hearing the ring. After that, when the single stimulation of the bell is adopted, the PSC will return to the threshold level (≈80 nA) after a relatively short relaxation time. Furthermore, mixing stimulation is used again to strengthen the effect of learning. It is obvious that the PSC is higher than that of the initial training and the salivation is triggered by the bell successfully after a longer relaxation time, which indicates the classical conditioning is well emulated based on our native oxide‐shelled GaSb NW synaptic transistor.

### 2.5 Mechanism Analysis of Native Oxide Shelled GaSb NWs FETs Characteristics

**Figure 5:**
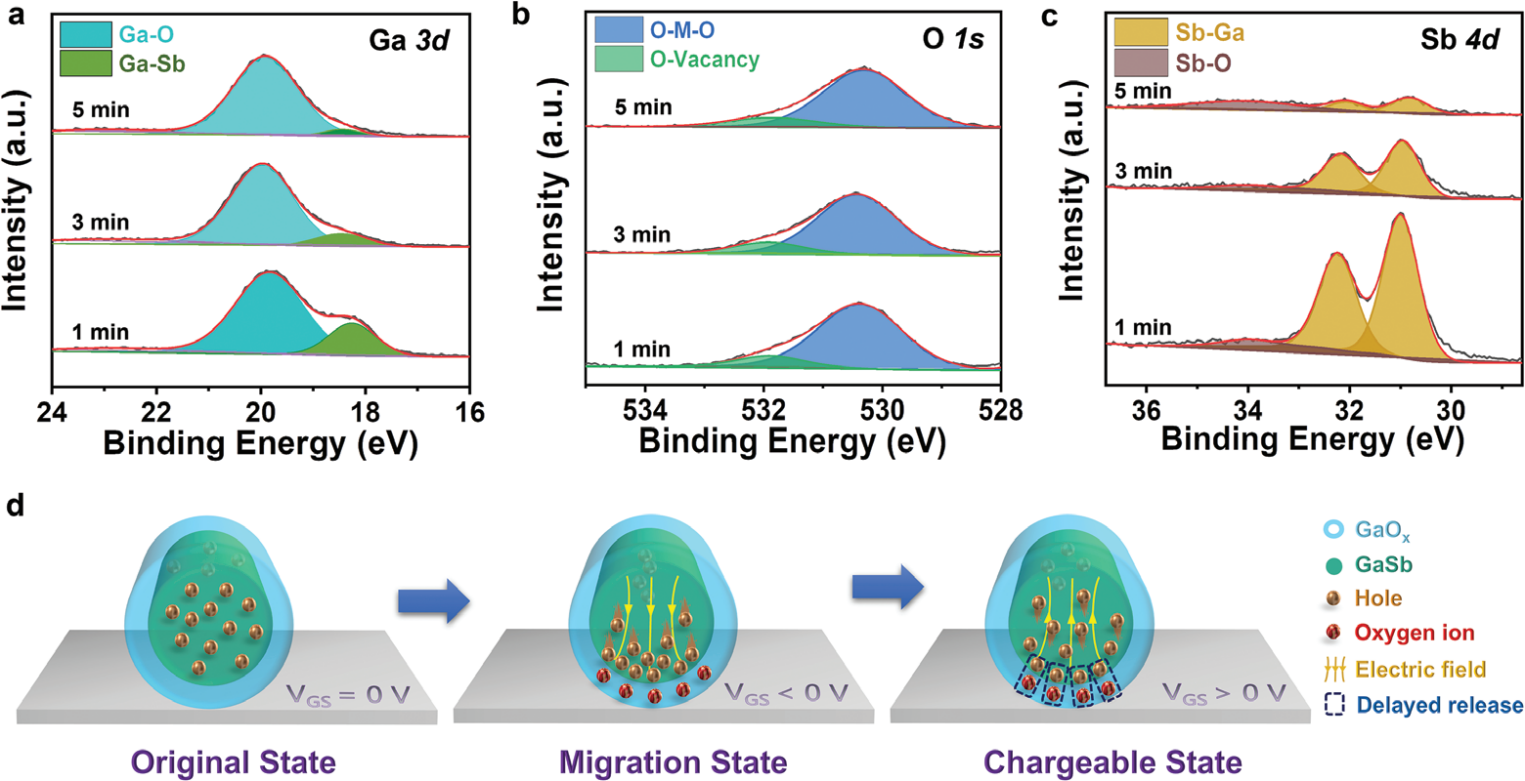
Mechanism analysis of native oxide‐shelled GaSb NWs FETs characteristics. XPS spectra of a) Ga 3*d*, b) O 1*s*, and c) Sb 4*d* of native oxide shelled GaSb NWs. d) Schematic illustration of the oxygen ions effect on the native oxide‐shelled GaSb NWs.

To explore the potential mechanism of native oxide‐shelled GaSb NWs FETs, X‐ray photoelectron spectroscopy (XPS) spectra are employed to monitor the compositional changes of the native‐oxide shelled GaSb NWs with different thermal annealing times. As well‐known that the penetration depth of X‐ray is <10 nm, thus, XPS mainly gives information on the top surface of the GaSb NWs.^[^ ^43^ ^]^ As shown in **Figure** **5**a, Ga 3*d* spectra are first deconvoluted into two Gaussian curves centered at ≈20.0 and ≈18.5 eV, corresponding to gallium oxide (Ga─O) and gallium antimonide (Ga─Sb) bond, respectively, without significant movement.^[^ ^33^ ^]^ The Ga─O content in the GaSb NWs steadily increases from 74.7% to 88.2% and 94.8%, as the thermal annealing oxidation time increases from 1 to 3 and 5 min, respectively. Obviously, the GaO*_x_* quantity increases and dominates in the surface of GaSb NWs along with the annealing process, which is in agreement with the above EDS elemental mapping results. To shed light on the detail of GaO*_x_*, O 1*s* spectra are performed with a low binding energy region of 530.4 eV peak and with a high binding energy region of 532.0 eV peak, corresponding to metal oxide (M‐O), and oxygen vacancy (oxygen ions), respectively (Figure 5b).^[^ ^44^ ^]^ Although the content ratio of oxygen vacancy increases slowly (from 11.5% to 13.6% and 14.3%) with the increase of annealing time (from 1 to 3 and 5 min), the huge increase in Ga─O content means the number of oxygen vacancies also increases significantly. It is noted that the Sb 4*d* spectra are observably weakened after annealing (Figure 5c), due to the Sb─O phase is not stable on the GaSb surface and may subsequently react with GaSb to form GaO*_x_* and elemental Sb.^[^ ^45^ ^]^ The above discussion indicates memory behaviors are from the oxygen vacancies (oxygen ions) of the amorphous GaO*_x_* shells of GaSb NWs, not SbO*_x_*.

As reported in the literature, the migration of oxygen ions in amorphous GaO*_x_* at room temperature usually causes wide hysteresis and memristive behavior.^[^ ^22^ ^]^ To further explore the generality of mobile oxygen ions, the pure GaO*_x_* NWs are generated based on GaSb NWs by high‐temperature processing. The typical *n*‐type transfer property and XRD patterns with completely amorphous spectra indicate the successful conversion of GaSb to amorphous GaO*_x_* (Figure S9, Supporting Information). The metal‐semiconductor‐metal structured device exhibits a bias tunable shape of the hysteresis *I–V* curves, ranging from narrow counter figure‐eight hysteresis (Figure S10, Supporting Information). This result indicates the mobile oxygen ions are present in this amorphous GaO*_x_* shell of GaSb NWs.^[^ ^22^ ^]^ In this case, as shown in Figure 5d, with a negative *V*_GS_, holes are accumulated from the original random uniform distribution state to be confined at the interface between the GaSb NWs and the dielectric. Meanwhile, the mobile oxygen ions in GaO*_x_* hop out of the equilibrium position and then migrate along the electric field in the reverse direction, accumulating near the GaSb outside surface. Consequently, the region near the dielectric is rich in oxygen vacancies. The non‐uniform distribution of oxygen ions will result in a *p*–*n*‐diode‐like structure, acting like a temporary chargeable floating gate. This chargeable effect suppresses and passivates the formation of hole trapping, causing an enhanced gate bias stress stability. Furthermore, upon *V*_GS_ changes to positive, part of the holes will quickly return to their original positions. However, another part of positively charged holes interacts with negatively charged oxygen ions, resulting in a slow‐release process of the chargeable state. Besides, the synaptic behavior of 5 min annealed GaSb NW FET under 200 K is explored the effect of mobile oxygen ions. As low temperature severely limits the diffusivity of oxygen ions, the charge‐trap‐induced *I–V* characteristic further verify that the distribution of oxygen ions and chargeable effect are the points to tune the electrical characteristics for mimicking the advanced synaptic behaviors and could be further enhanced by improving the quality of the oxide shells.

## 3 Conclusion

In summary, the native surface oxides of III–V semiconductors usually act as charge trapping centers, leading to the unwanted oxide‐semiconductor interfacial defects and poor stability of as‐constructed electrical devices. Here, the bias‐stress stability and reliable GaSb NW synaptic FETs are developed by purely manipulating the native oxide shell of GaO*_x_*. The amorphous native GaO*_x_* shell on GaSb NWs could be well controlled via an expeditious, tunable in‐situ thermal annealing process. The optimal 5 min annealed GaSb NW FET exhibits excellent stability with a minimal *V*_th_ shift of ≈0.54 V (calculated newly generated trap density of ≈2.4 × 10^11^ cm^−2^) under 60 min voltage bias of *V*_GS_ = −10 V and *V*_DS_ = 0.4 V, which is >15× stability than the pristine GaSb NW FET. The enhanced gate bias‐stress stability is benefited from the mobile oxygen ion in the native GaO*_x_* shell, strongly reducing the trapping density. Furthermore, the artificial synaptic chargeable‐dielectric free FET is demonstrated utilizing this presented native GaO*_x_*‐shelled GaSb NWs. This native oxide shell maintains the quality of the core NWs channel and reduces the manufacturing cost. Various synaptic functions, such as STP, LTP, and advanced neural Pavlovian conditioning are successfully mimicked in this synaptic FET. The oxygen ions migration in the amorphous GaO*_x_* shell under sweep voltage will cause a chargeable effect, resulting in tunable synaptic behaviors. This work provides a simple and efficient approach to utilizing native oxide shells of semiconductors for low‐cost and stable neuromorphic computing systems.

## 4 Experimental Section

### GaSb NWs Growth and Devices Fabrication

GaSb NWs studied in this work were grown by surfactant‐assisted solid‐source CVD method in a dual‐zone horizontal tube furnace.^[^ ^46^ ^]^ In short, the catalyst of Au film (1 nm thick) was deposited on the 50 nm thick SiO_2_ substrate by using thermal evaporation. The source material of GaSb powder (99.999% purity) was placed in the upstream zone. Besides, the surfactant of sulfur (99.99% purity) was located in the middle of two zones and the Au film was located in the middle of the downstream zone. Next, the pressure of the growth chamber was pumped down to 6 × 10^−3^ Torr by a mechanical pump. During the NWs growth, the source was heated to 750 °C, while the substrate was heated to 560 °C with the flow rate of the carrier gas of 200 sccm hydrogen (99.9995% purity) for 25 min. When the growth was completed, the heating of the source and substrate were stopped together and the as‐prepared GaSb NWs could be taken out as the furnace was naturally cooled down to room temperature under the hydrogen flow. The pure GaO*_x_* NWs were generated based on GaSb NWs by 700 °C thermal oxidating for 10 min in the atmosphere by horizontal tube furnace. The as‐prepared NWs were suspended in ethanol solution by ultrasonication and then drop‐cast onto the highly doped *p*‐type Si substrates with a 50 nm thick SiO_2_ layer, followed by annealing on a 300 °C hot plate for 0, 1, 3, and 5 min (RH ≈ 30%). Next, the source/drain regions of FETs were defined by UV photolithography, and 50 nm thick Ni film was deposited by *e*‐beam evaporation as the contact electrode. After a lift‐off process, the location of the as‐fabricated back‐gated NW FET would be checked by microscope.

### Material Characterization and Electrical Characterization

The morphologies of the as‐prepared NWs were characterized by SEM (Helios G4 UC, Thermo Scientific) and TEM (JEM‐F200, JEOL). HRTEM and HAADF‐STEM associated with EDS elemental mapping were performed at 300 kV on an aberration‐corrected transmission electron microscope equipped with a Super‐X EDS detector (Spectra 300, Thermofisher). The crystal phase and phase purity of as‐prepared NWs were verified by XRD (D8 Advance, Bruker). The chemical composition and elemental valence were analyzed by XPS (Nexsa, Thermo Scientific). The transfer and output characteristics of the fabricated devices were measured at room temperature using an Agilent B1500A semiconductor parameter characterization system. The *V*_th_ was extracted by linear extrapolation method from the forward‐sweep transfer curve. The *I*_on_ represents the on‐current of NWs FETs under *V*_GS_ = −10 V. The carrier mobility was calculated by using the equation of *µ* = *g*×(*L* ^2^/*C*_OX_)×(1/*V*_DS_). *g* is transconductance, which could be extracted from the transfer curve following the equation of *g* = (d*I*_DS_)/(d*V*_GS_)|*V*_DS_. *C*_OX_ is the gate capacitance obtained by finite element analysis software COMSOL.^[^ ^31^ ^]^ In gate bias‐stress stability tests, the transfer characteristics were measured at the indicated time and then proceed to the continuous bias condition. A Keithley 2602B semiconductor characterization system was used to assess the electrical performance and PSC in response to applied electrical pulses. The periodic optical signal was generated by the waveform generator controlling the power input of the 785 nm diode laser. All electrical measurements of GaSb NWs FETs were carried out under a relatively low humidity of ≈30% RH. Temperature‐dependent experiments were carried out under high vacuum (≈10^−5^ Torr) at 200 K using a cryogenic probe station (LakeShore TTPX).

### Statistical Analysis

Quantitative data, such as hysteresis statistics of Figure 2c,d and performance metrics statistics of Table 1 were expressed as mean ± standard deviation. At least ten samples were used for each condition for statistics. All data processing and analyses were obtained by using the software of Origin 2021 and Excel 2019.

## Conflict of Interest

The authors declare no conflict of interest.

## Supporting information

Supporting Information

## Data Availability

The data that support the findings of this study are available from the corresponding author upon reasonable request.
