# Supplementary material for: An Amorphous Native Oxide Shell for High Bias‐Stress Stability Nanowire Synaptic Transistor
Source: Adv Sci (Weinh). 2023 Sep 28;10(31):2302516. doi: 10.1002/advs.202302516 (PMC10625101; doi:10.1002/advs.202302516)
Supplement: Supplementary file 1 — Supporting Information [file ADVS-10-2302516-s001.pdf]

ADVANCED SCIENCE
Open Access

Supporting Information

for *Adv. Sci.*, DOI 10.1002/advs.202302516

An Amorphous Native Oxide Shell for High Bias-Stress Stability Nanowire Synaptic Transistor

*Xinming Zhuang, Zixu Sa, Jie Zhang, Mingxu Wang, Mingsheng Xu, Fengjing Liu, Kepeng Song, Tao He, Feng Chen and Zai-xing Yang**

Supporting Information

An Amorphous Native Oxide Shell for High Bias-stress Stability Nanowire Synaptic Transistor

Xinming Zhuang,[†] *Zixu Sa,*[†] *Jie Zhang, Mingxu Wang, Mingsheng Xu, Fengjing Liu, Kepeng Song, Tao He, Feng Chen, Zai-xing Yang**

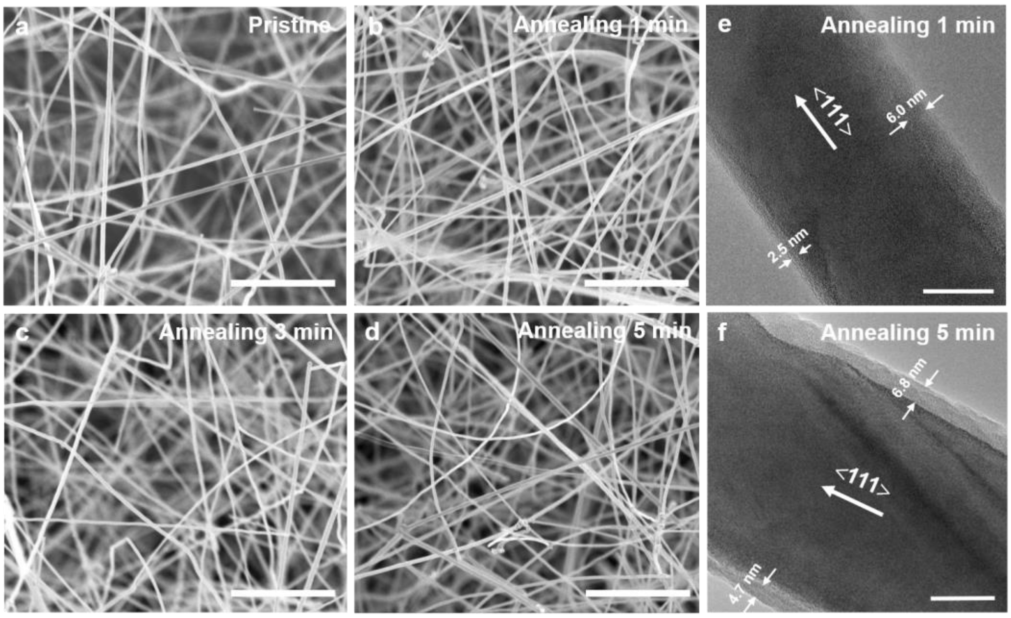

Figure S1. The SEM images of (a) pristine, (b) annealing 1 min, (c) annealing 3 min and (d) annealing 5 min GaSb NWs. HRTEM images of (e) annealing 3 min and (f) annealing 5 min samples in this study. The scale bars of a-d and e-f are 1 μm and 10 nm, respectively.

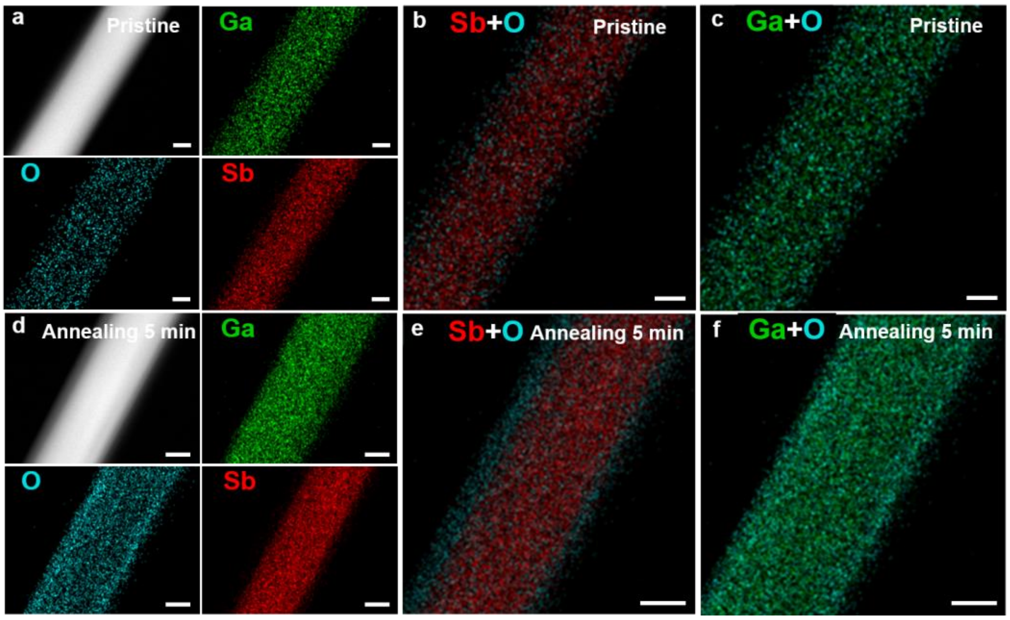

Figure S2. (a-c) EDS elemental mapping images of individual element (Ga, O, and Sb), Sb+O elements, and Ga+O elements, respectively, of pristine sample. (d-f) EDS elemental mapping images of individual element (Ga, O, and Sb), Sb+O elements, and Ga+O elements, respectively, of annealing 5 min sample. All the scale bars are 20 nm.

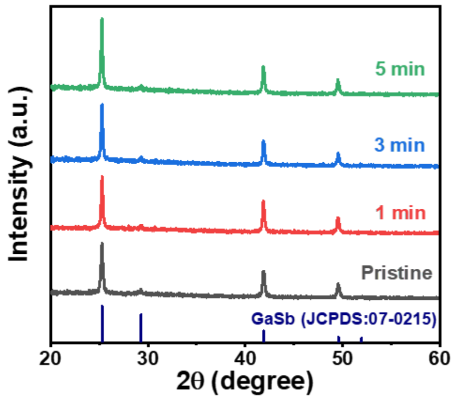

Figure S3. XRD patterns of native oxide shelled GaSb NWs under indicated annealing times.

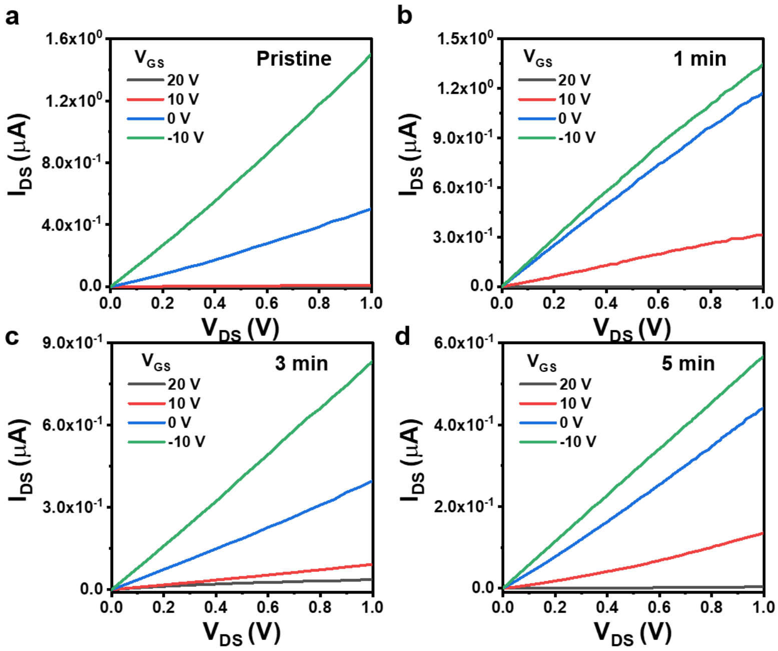

Figure S4. Representative output characteristics for the FETs based on GaSb NWs with various annealing times.

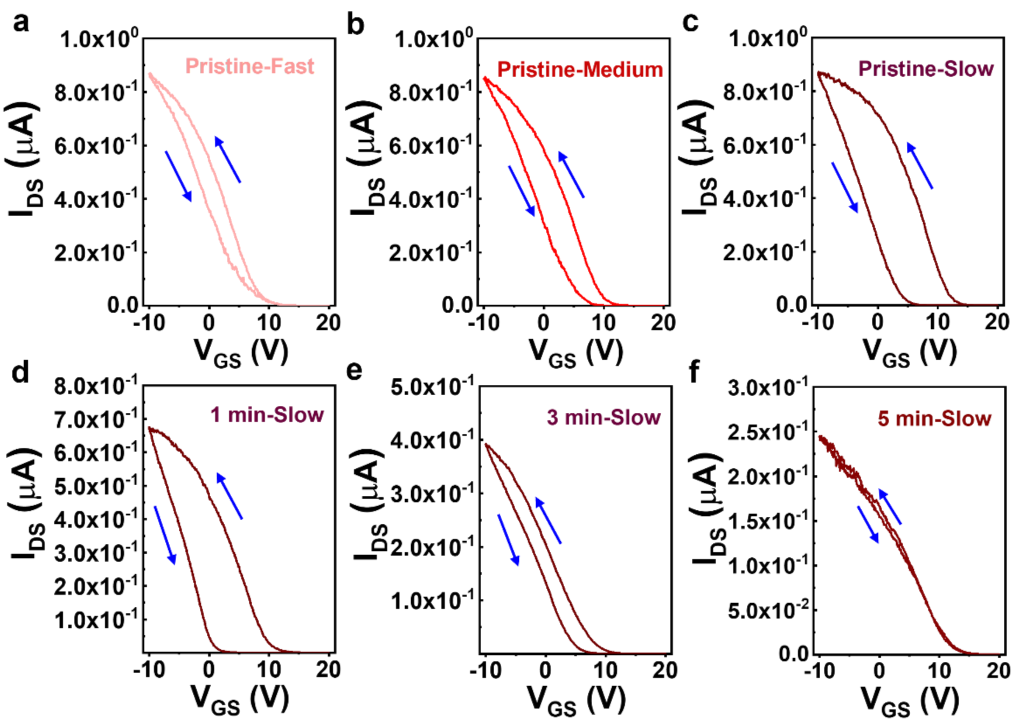

Figure S5. Representative transfer characteristics for the pristine GaSb NWs FETs with various scanning rates of (a) fast, (b) medium, and (c) slow under V_{DS} = 0.4 V. (d-f) Representative transfer characteristics for the FETs based on GaSb NWs with various annealing times with slow scanning rates under V_{DS} = 0.4 V.

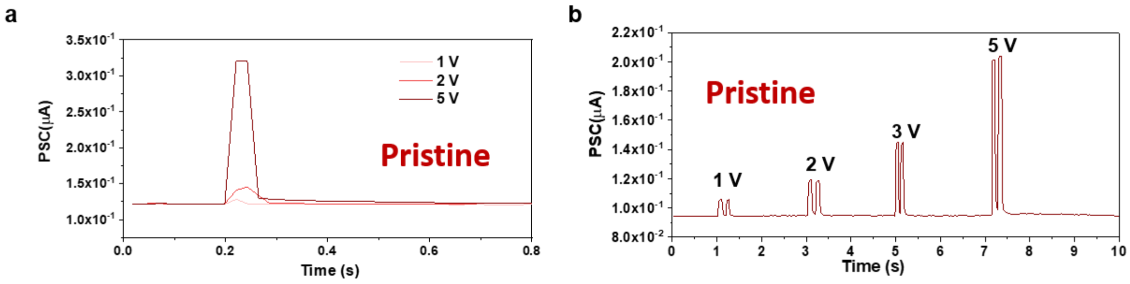

Figure S6. The pristine device synaptic characteristics when the presynaptic spike increased from -1 to -5 V with a period of 100 ms.

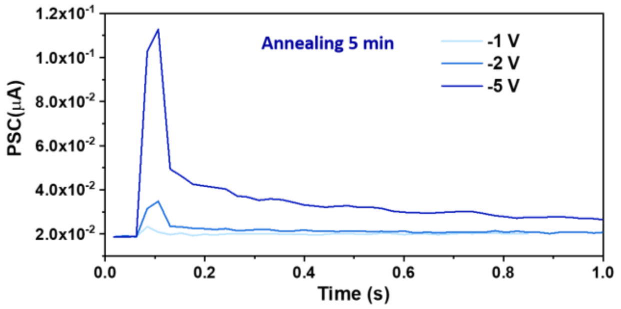

Figure S7. The synaptic characteristics of annealing 5 min device of when the presynaptic spike increased from -1 to -5 V with a period of 100 ms.

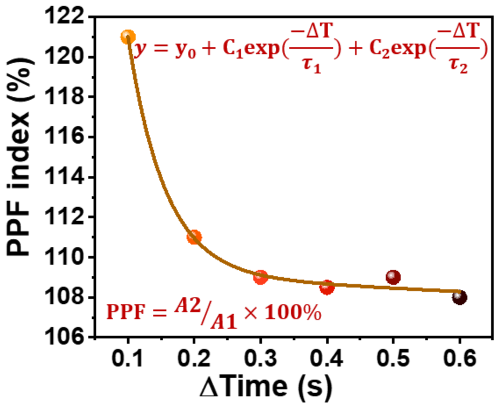

Figure S8. Corresponding PPF index as a function of different pulse intervals.

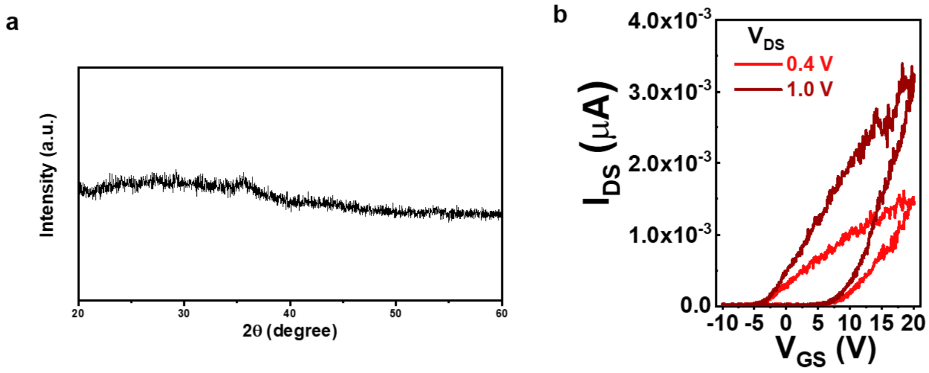

Figure S9. a, XRD pattern of GaO_x NWs. b, Representative transfer characteristics for the FET based on GaO_x NW.

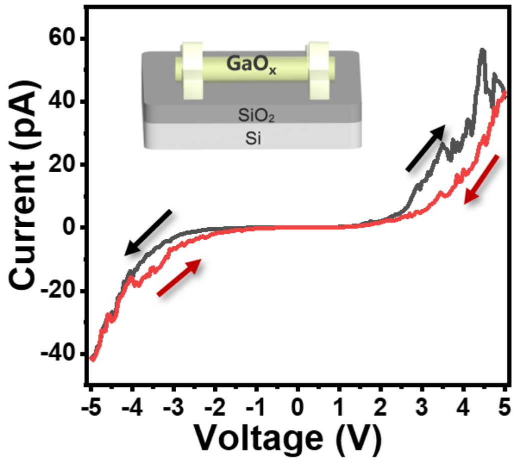

Figure S10. I-V characteristics of GaO_x NW voltages weep cycle of 0→+2→-2→0 V at a sweep rate of 1.1 $V{\cdot}s^{-1}$.

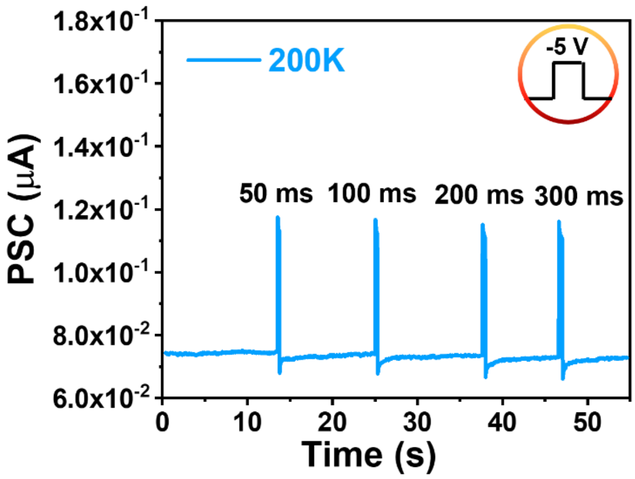

Figure S11. Synaptic behaviors of the 5 min annealed GaSb NW FET at V_{DS} = 0.4 V for temperatures under 200 K.
